# Dietary biogenic selenium nanoparticles improve growth and immune-antioxidant indices without inducing inflammatory responses in Nile tilapia

**DOI:** 10.1038/s41598-024-72022-w

**Published:** 2024-09-23

**Authors:** Ahmed H. Al-Wakeel, Samia Elbahnaswy, Elsayed A. Eldessouki, Engy Risha, Eman Zahran

**Affiliations:** 1 Department of Aquatic Animal Medicine, Faculty of Veterinary Medicine, Mansoura University, Mansoura, 35516 Egypt; 2 Department of Fish Health and Diseases, Faculty of FishResources, Suez University, Suez, Egypt; 3 Department of Clinical Pathology, Faculty of Veterinary Medicine, Mansoura University, Mansoura, 35516 Egypt

**Keywords:** *Oreochromis niloticus*, Nanoparticles, Health biomarkers, Histopathology, Immunity, Nanobiotechnology, Cytokines, Innate immunity, Animal physiology

## Abstract

The present study evaluated the use of green-synthesized selenium nanoparticles (SeNPs), using the microalgae *Pediastrum boryanum* as a diet additive in aquaculture to improve the growth performance, health, and immune response of Nile tilapia. Nile tilapia were fed different concentrations of green SeNPs (79.26 nm) as follows: 0, 0.75, and 1.5 mg/kg of SeNPs for 8 weeks. Following the trial, growth performance, biochemical indices, antioxidant and pro-inflammatory cytokine-related genes, and tissue histological examinations were performed. The study showed that SeNPs significantly improved (*P* < 0.05) growth performance and innate immune parameters (*P* < 0.001, IgM, and lysozyme) at both supplemented doses compared with the control. The protein profile and liver function enzymes were normal compared with those in the control group (*P* > 0.05). Serum malondialdehyde and superoxide dismutase levels were not significantly changed, while reduced glutathione and catalase were significantly enhanced (*P* < 0.01, *P* < 0.05) in the SeNPs 1.5 mg/kg compared to the control group. No inflammatory response was detected upon SeNP supplementation, as indicated by the absence of changes in the expression of pro-inflammatory cytokine genes. The earlier assays’ results were histopathologically evidenced, where hepatic and splenic tissue architectures in SeNPs groups did not reveal any deviation from the control group. Our findings indicate that green selenium nanoparticles can potentially improve the growth and immunological response of Nile tilapia, offering opportunities for incorporating health benefits into functional foods and nutraceuticals, which corresponds to the increasing consumer interest in eco-friendly, environmentally sustainable dietary supplements.

## Introduction

Aquaculture production has diversified extensively to meet the rising global demand for aquatic foods, with a major research focus on farming important finfish species, such as Nile tilapia (*Oreochromis niloticus*), through ongoing scientific advancements and innovation in sustainable aquaculture practices^1,2^. Global aquaculture of Nile tilapia has been steadily increasing over the years. In 2018, the world’s aquaculture food production reached a high record of 82.1 million tonnes, with Asia leading global production^3^. The sustainability of global aquaculture practices is a concern, with the overall sustainability score being low and none achieving a high sustainability score^4^. Therefore, developing and utilizing alternative sustainable feed sources with high nutritional values is essential for advancing environmentally and socially responsible aquaculture practices.

Nanoparticles are used as feed additives in aquaculture to improve the growth performance, health, and immune responses of fish species^5–8^. Green synthesized nanoparticles from algae offer potential benefits in terms of providing more effective fish feed, enhancing the availability of micronutrients, and promoting faster development in fish^9,10^. Several non-metallic and metallic nanoparticles, including selenium and zinc, have been studied for their positive effects on fish growth^11,12^. However, green NPs synthesized using algae improve the availability of micronutrients in feed, leading to better growth and immune responses in aquatic organisms^6,13^. Additionally, nanoparticles have the potential to be used as novel fish growth promoters, vaccines, and drug delivery agents in aquaculture^8^. Overall, nanotechnology has the potential to improve fish health, disease control, and sustainability in the aquaculture industry.

Of particular interest, selenium nanoparticles (SeNPs) synthesized using green methods have been studied for their potential applications in aquaculture, as they have shown positive effects on the growth and immunity of fish and have been found to reduce the harmful effects of heavy metals and bacterial load in fish, leading to improved fish gut health, growth, and behavior^14^. They also exhibit antimicrobial activity against important fish and crustacean pathogens, such as *Vibrio harveyi* and *Vibrio parahaemolyticus*^15^. The application of selenium nanoparticles in aquafeeds has been shown to improve growth performance, physiology, antioxidant capacity, and immune response^16^. Additionally, green-synthesized selenium nanoparticles have been found to effectively control aquatic pathogens, including those with multidrug resistance, making them a potential substitute for conventional antibiotics in aquaculture^17^. Supplementing aquaculture diets with biologically synthesized nano-selenium can mitigate oxidative stress and increase stress resistance and productivity of farmed aquatic organisms^18^. We examined the influence of SeNPs on selected key parameters, including growth performance, serum biochemical and immunological parameters, antioxidant status, and oxidative stress markers, as well as on hepatic gene expression and histopathological examination of Nile tilapia. Our groundbreaking research focuses on the innovative green synthesis of SeNPs. We employ a new microalgal extract, *P. boryanum*, producing higher levels of secondary metabolites besides other unique properties. Our study involves a highly dominant species, Nile tilapia, crucial for advancing its aquaculture practices. This holistic approach offers a comprehensive understanding of how green synthesized SeNPs impact fish health.

## Materials and methods

### Source of green synthesized SeNPs and Dietary preparation

The microalgae, *P. boryanum* (Powder), was purchased from the National Research Center in Cairo, Egypt, and was used as a capping agent to greenly synthesized selenium nanoparticles (SeNPs) according to our recent study^19^ (Supplementary file). Briefly, Aan adaptation methodology^20^ was used to aqueous extract the active components from the microalgae, *P. boryanum,* following a previous report^21^. Distilled magnetized water was used to extract the algal powder at 70 °C for 2 h. Se (IV) oxide was used to produce nanoparticles. The algal extract was mixed with a one mmol aqueous solution of Se salt and agitated for 2 h at room temperature. The mixture was then exposed to UV radiation for 20 min, and the created nanoparticles were filtered and stored at − 18 °C for further use. The Se characterization was conducted through several assays, including UV–Vis spectroscopic analysis (PG instruments T80 + UV/vis spectrometer, UK), FTIR spectroscopy (Nicolet iS10 FT-IR spectrometer (Thermo Scientific, USA), Transmission Electron Microscope (TEM) (JEOL TEM-2100, Tokyo, Japan), X-ray Diffraction, and Zeta-Potential (Kassel, Germany)**.** The obtained SeNPs were within the range of 72.16–89.45 nm. Three distinct regimens were formed as described elsewhere^22^, based on the initial diet, using doses of 0, 0.75, and 1.5 mg/kg, and were referred to as follows. Control (a basal diet containing Se in the mineral mix (inorganic form Na_2_Seo_3_ as 0.2 mg/Kg diet), SN1, and SN2 (fed a diet contained Se-free mineral premix and replaced with SeNPs at 0.75 and 1.5 mg/kg body weight), correspondingly. The fish feed components were prepared following the guidelines of the NRC^23^ (Supplementary Table 1). All ingredients were mixed with gelatin, and water was added until stiff dough was formed. The paste was pelleted into 3-mm-diameter pellets using a meat mincer (ME605131 1600-Watt, Moulinex, Groupe SEB, France). The resulting strands were shadow-dried, broken up, sieved into pellets, and stored in plastic bags at 4 °C until use.

### Experimental fish and diets

A total of 180 Nile Tilapia, each with an average body weight of 57.5 ± 2.5 g, were selected for the study and assigned to three different experimental groups in a private fish farm in Manzala, Dakahlia governorate, Egypt. Each fish group (in triplicate) contained 20 fish/hapa, each measuring 200 × 500 × 100 cm^3^ (10 m^3^). The water quality parameters were in a temperature range of ~ 26 °C, pH 7–8, and DO > 5 mg/L. The fish groups SN0, SN1, and SN2 were fed twice daily at 09:00 and 15:00, with the feed amounting to 3% of fish biomass; fish were weighed every 2 weeks, and the feeding rate was adjusted accordingly. The feeding trial lasted for 8 weeks.

### Growth performance assessment

Three fish from each hapa (Total number of fish sampled per group, N = 9) were sampled upon completion of the trial. Each aquarium was sampled one at a time; the three fish were sedated at 30 mg/L of buffered tricane; (MS-222^®^ARGENT). Each fish was weighed and measured to calculate the final body weight (FBW), length, and Condition factor (K) = (W/L^3^) × 100; where: W = weight of fish in grams and L = total length of fish in “cm.”

### Samples collection

Three fish from each hapa (N = 9) were sampled, and each fish was euthanized one at a time in a separate container with a high dose of buffered tricane (200 mg/L). Blood samples were collected from caudal vessels. The collected blood samples were placed in sterile tubes and allowed to clot at room temperature for 4 h before centrifugation at 1198×*g* for 10 min to obtain serum for immunological and biochemical analyses and evaluation of oxidant/antioxidant activities. Immediate dissection was performed, and the liver and spleen were weighed to calculate the hepatosomatic index (HSI) and spleen-somatic index (SSI), respectively, using the following formulae:

HSI = Wt of fish liver/fish BWt × 100*;* SSI = Wt of fish spleen/fish BWt × 100*.* The liver was split into two portions, the 1st portion was preserved in RNAlater^®^ (Sigma Aldrich, USA) for subsequent immune gene expression analysis. The 2nd liver portion and spleen were fixed in 10% neutral-buffered formalin for histopathological examination.

### Evaluation of serum biochemical and immunological parameters

Serum biochemical parameters, including protein profile (total protein and albumin), were measured using commercial kits (VitroScient, Egypt, and Germany), and the activities of serum alanine aminotransferase (ALT) and aspartate aminotransferase (AST) were measured using commercial kits (Spinreact, Spain). Measurements were performed using a 5010 Photometer (BM Co., Germany) according to the manufacturer’s instructions. Immunological parameters, including serum lysozyme activity (LZM), IgM levels, and LZM activity, were quantified using fish lysozyme ELIZA kits (MyBioSource, Inc., USA), following the manufacturer’s guidelines. The optical density was measured at a wavelength of 450 nm. The lysozyme concentration in the samples was then calculated by comparing the optical density of the samples to a standard curve, and the results were reported in µg/mL. Serum IgM levels were determined using fish IgM ELIZA kits (CSBE-12045Fh, CUSABIO BIOTECH CO., Ltd., China) according to the manufacturer’s instructions. Optical density was measured at 450 nm. The IgM concentration in the samples was determined by comparing the optical density of the samples to the standard curve, and the results were reported in µg/mL.

### Determination of serum antioxidant status and oxidative stress markers

Spectrophotometric measurements 5010 Photometer (BM Co., Germany) of serum malondialdehyde (MDA), reduced glutathione (GSH), superoxide dismutase (SOD), and catalase (CAT) activities were conducted using a colorimetric method on a 6745 UV/Vis spectrophotometer, following the manufacturer’s instructions for the kits (Bio-diagnostics, Egypt), used to measure MDA, GSH, and SOD levels at 534, 405, and 560 nm, respectively, as outlined in previous studies^24,25^. These levels were expressed as nmol/L serum. CAT activity was determined using an Elabscience^®^ biochemical assay kit (Elabscience Biotechnology Inc., USA) following the method described by Aebi^26^, which involves measuring the reduction in hydrogen peroxide concentration at 240 nm, with results expressed as U/L serum.

### Expression of the immune-related genes

Approximately 100 mg of liver tissue was used for total RNA extraction using Genzol™ (Geneaid Biotech Ltd, Taiwan) and a manual homogenizer without DNase treatment. The resulting pellet was dissolved in TE buffer (pH 8.0) as described elsewhere^27^. The RNA concentration was calculated using a Nanodrop spectrophotometer (Q5000/Quawell, Massachusetts, USA). To synthesize complementary DNA (cDNA), 1 µg of total RNA was utilized using a TOPscript™ RT DryMIX(dT18) cDNA Synthesis Kit (Enzynomics Co Ltd, Daejeon, Republic of Korea) following the manufacturer’s instructions. To amplify selected genes of Nile tilapia, including the stress marker/gene heat shock protein-70 (*HSP-70*), proinflammatory gene/tumor necrosis factor-alpha (*TNF-α*), anti-inflammatory genes/transforming Growth Factor-β (*TGF-β1*) and Interleukin-10 (*IL-10*), along with beta-actin (*β-actin*) as a housekeeping gene, specific primers were used (Table 1) were used, as previously reported^28^. The QuantStudio1™ Real-Time PCR System (Applied Biosystems™ Thermo Fisher Scientific, USA) was used to analyze gene expression using the Solg™ 2X Real-Time PCR Smart mix (SolGent Co., Ltd. Yuseong-gu, Daejeon, Korea). The thermocycling conditions were set at 95 °C for 20 s, followed by 40 cycles of denaturation at 60 °C for 40 s and elongation at 72 °C for 30 s. Relative gene expression levels were evaluated in triplicate included on template controls using the 2^−ΔΔCT^ formula^29^.

**Table 1:** Primers used for gene expression using real-time PCR analysis.

Gene	Forward (5′–3′)	Reverse (5′–3′)	Size (bp)	Accession number
TNF-α	GAATACAAGGCCAGAAAGGATGACAC	GACCTTTTGAGTCGCTGCCTTC	155	NM_001279533
TGF-β1	CAGGAAAGATCTAGGATGGAAGTGGA	TGGACAGCTGCTCGACCTTGTG	236	XM_003459454
HSP70	TTCAAGGTGATTTCAGACGGAG	CTTCATCTTCACCAGGACCATG	111	XR_002061784.2
IL-10	CTGCTAGATCAGTCCGTCGAA	GCAGAACCGTGTCCAGGTAA	94	XM.003441366.2
Β-actin	GGGAGAAGATGACCCAGATCATGT	CCTCGTAGATGGGCACTGTGTG	156	XM_003443127.5

### Histopathological examination

Liver and spleen tissue samples were preserved in 10% neutral buffered formalin for 24 h, embedded in paraffin wax, and sectioned at a thickness of 5 µm. To examine their morphology and integrity, Hematoxylin and Eosin (H&E) staining was uniformly applied to specific slides following the protocol detailed by Suvarna et al.^30^. Histomorphometric measurements were obtained by scrutinizing the stained slides under a light microscope (Olympus CX 31) and capturing images with an attached camera (Olympus DP 21 digital camera) (Olympus Corporation, Tokyo, Japan), as previously described^31^.

### Statistical analysis

Before one-way analysis of variance (ANOVA), tests for normality and homogeneity were conducted using the Kolmogorov–Smirnov and Levene’s tests, respectively. Analyses were performed using GraphPad^®^ statistics package version 8.4.2. (GraphPad Software, Inc., USA). Tukey’s honestly significant difference test was used to investigate the differences between means. The thresholds for significance were established at *P* < 0.05 (*), *P* < 0.01 (**), and *P* < 0.001 (***). All data are expressed as mean ± SE.

## Results

### Growth performance assessment

**Fig. 1:**
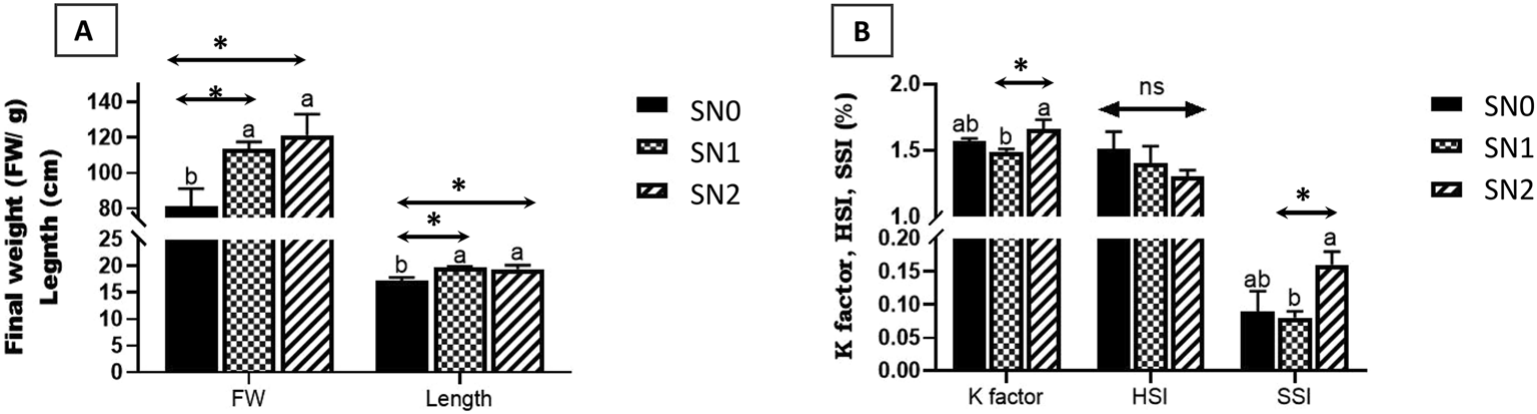
Growth indices of Nile tilapia supplemented with green synthesized selenium nanoparticles (SeNPs) at 0, 0.75, and 1.5 mg SeNPs/kg for 8 weeks (N = 9). Data were expressed as Mean ± SEM. Values with a different letter superscript are significantly different between groups (One-way ANOVA with post-hoc Tukey test, *P* < 0.05; *P* < 0.01; *P* < 0.001). Asterisks indicate levels of significance (ANOVA with post hoc Tukey test, *P < 0.05; **P < 0.01; ***P < 0.001).

The bar graphs in Fig. 1 illustrate the growth performance parameters of the fish groups. The final body weight (FW) and length presented in Fig. 1A were in the same pattern, where they significantly increased (*P* < 0.05) in SN1 (FW: 113.75 ± 3.75, Length: 19.7 ± 0.24) and SN2 (FW: 121.25 ± 11.97, length: 19.375 ± 0.8) fish groups compared to the SN0 group (FW: 81.25 ± 9.87, length: 17.225 ± 0.63). The K factor and SSI were also in the same pattern, whereas SN2 fish groups (K factor: 1.66 ± 0.07, SSI: 0.16 ± 0.02) showed higher values (*P* < 0.05) than the SN1 fish group (K factor: 1.49 ± 0.02, SSI: 0.08 ± 0.01) with insignificant values of both SN1 and SN2 compared to the SN0 group (K factor: 1.57 ± 0.02, SSI: 0.09 ± 0.03). HSI showed no significant differences (*P* > 0.05) across all groups (Fig. 1B).

### Evaluation of serum biochemical and immunological parameters

**Fig. 2:**
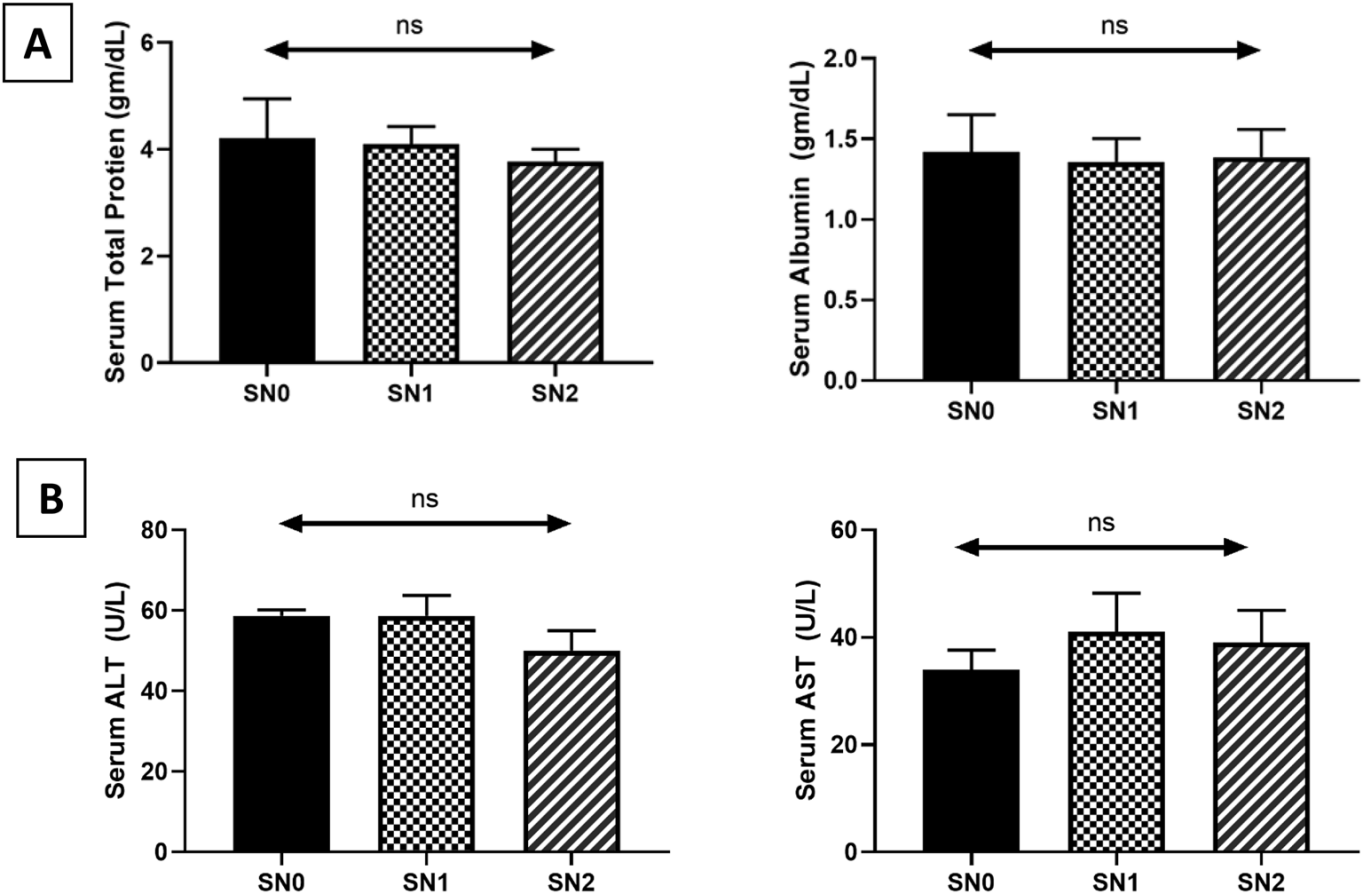
Protein profile (total protein, albumin, and globulin) and enzyme activities (ALT and AST) of Nile tilapia supplemented with green synthesized selenium nanoparticles (SeNPs) at 0, 0.75, and 1.5 mg SeNPs/kg for 8 weeks (N = 9). Data were expressed as Mean ± SEM. Values with a different letter superscript or none indicate significant or insignificant differences between groups. (One-way ANOVA with post-hoc Tukey test, *P* < 0.05; *P* < 0.01; *P* < 0.001).

**Fig. 3:**
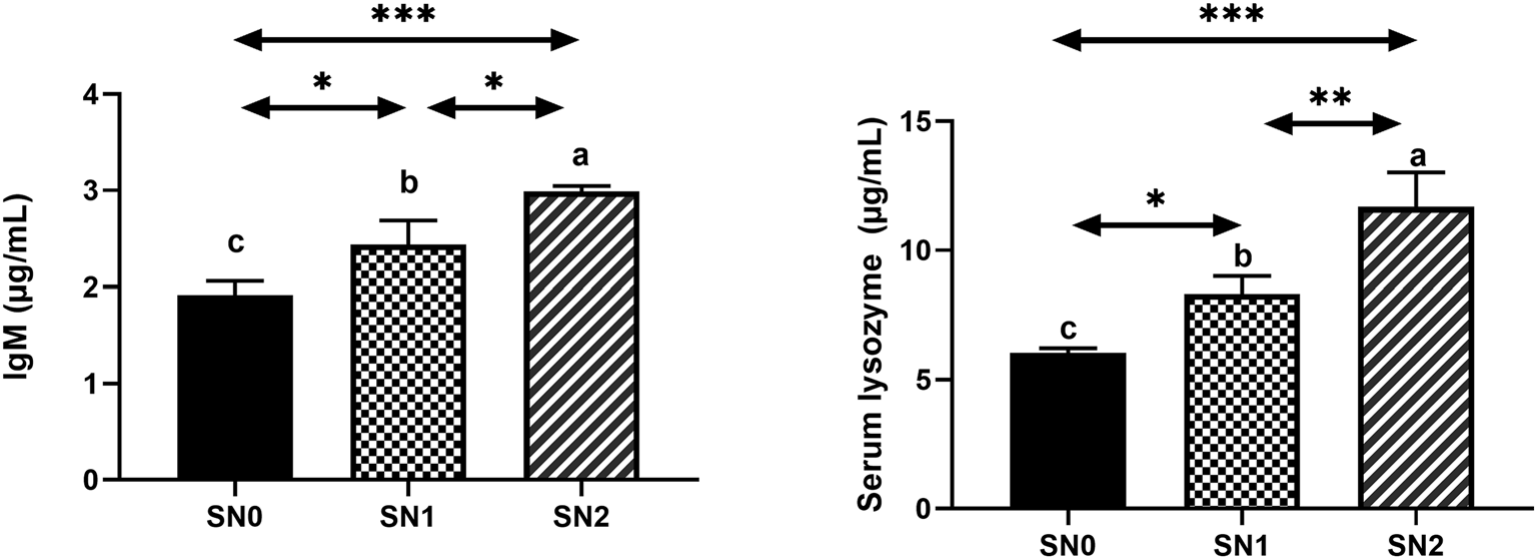
Lysozyme (LZM) and IgM of Nile tilapia supplemented with green synthesized selenium nanoparticles (SeNPs) at 0, 0.75, and 1.5 mg SeNPs/kg for 8 weeks (N = 9). Data were expressed as Mean ± SEM. Values with a different letter superscript are significantly different between groups. Asterisks indicate levels of significance (ANOVA with post hoc Tukey test, *P < 0.05; **P < 0.01; ***P < 0.001).

Serum biochemical and immunological parameters are presented in Fig. 2A,B. The protein profile (total protein and serum albumin levels), ALT, and AST functional enzymes confirmed the safety of using SeNPs on liver health status, as reflected by insignificant changes compared with the control. Immunological parameters, including IgM and LZM levels, showed similar patterns (Fig. 3). A notable increase in IgM levels was observed in the SN1 (2.44 ± 0.14, *P* < 0.05) and SN2 (2.99 ± 0.03, *P* < 0.001) fish groups compared to that in the SN0 group (1.91 ± 0.09), with a statistical difference (*P* < 0.05) between the former. Similarly, the LZM level exhibited a significant increase in the SN1 (8.31 ± 0.40, *P* < 0.05) and SN2 (11.7 ± 0.7, *P* < 0.001) fish groups compared to the SN0 group (6.05 ± 0.09), with a statistical difference (*P* < 0.05) between the former.

### Determination of antioxidant status and oxidative stress markers

**Fig. 4:**
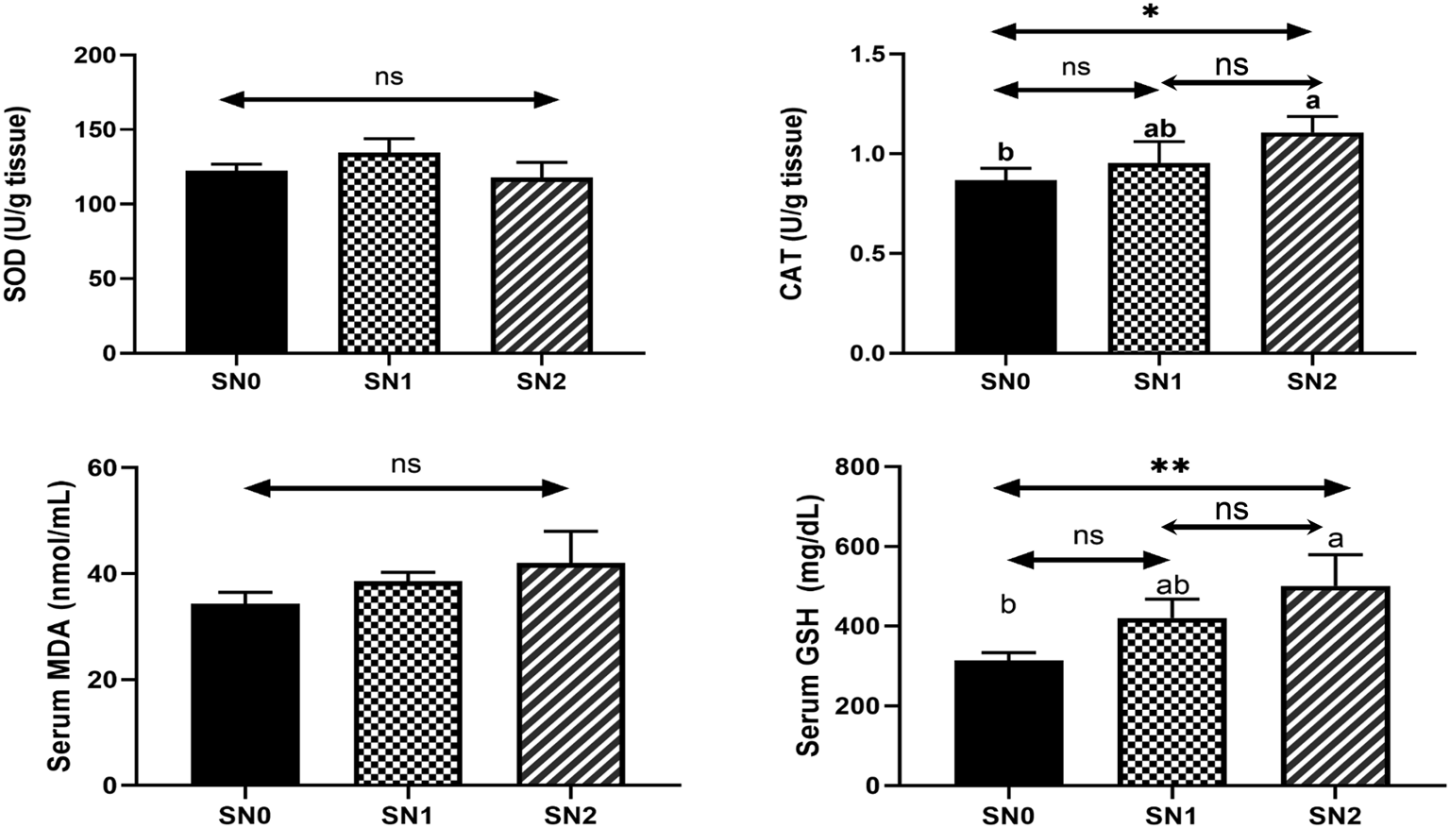
Oxidant (malondialdehyde/ MDA) and antioxidant (catalase/CAT, glutathione reductase/ GSH, and Superoxide dismutase/SOD) parameters of Nile tilapia supplemented with green synthesized selenium nanoparticles (SeNPs) at 0, 0.75, and 1.5 mg SeNPs/kg for 8 weeks (N = 9). Data were expressed as Mean ± SEM. Values with a different letter superscript are significantly different between groups. Asterisks indicate levels of significance (ANOVA with post hoc Tukey test, *P < 0.05; **P < 0.01; ***P < 0.001).

The oxidant/antioxidant parameters are shown in Fig. 4. The MDA and SOD levels were not significantly different among the groups. The CAT and GSH levels displayed a similar pattern, where they were significantly increased in the SN2 fish group (CAT/1.11 ± 0.05, *P* < 0.05, GSH/501 ± 39.1, *P* < 0.01) compared to the SN1 (CAT/0.95 ± 0.06, GSH/420 ± 23.9) and SN0 (CAT/0.87 ± 0.03, GSH/314 ± 9.88) fish groups.

### Gene expression of Hepatic mRNA level of Nile tilapia

**Fig. 5:**
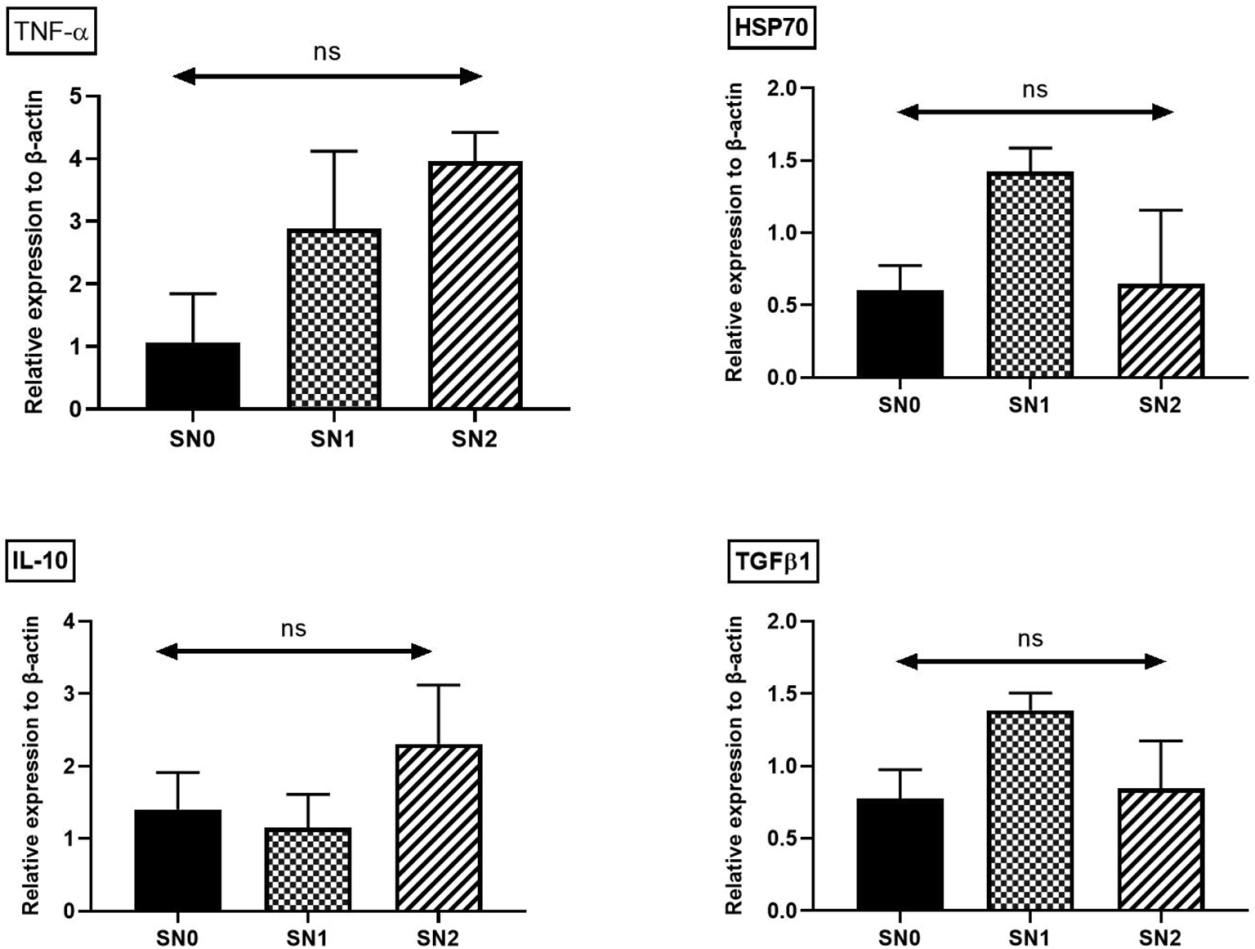
Comparative hepatic gene expression of (**A**) pro-inflammatory genes (tumor necrosis factor-α/ TNF-α,), and anti-inflammatory gene (interleukin-10/ IL-10 and transforming growth factor/TGF-β1), and (**B**) stress-related genes (heat shock protein70/HSP70) of Nile tilapia supplemented with green synthesized selenium nanoparticles (SeNPs) at 0, 0.75, and 1.5 mg SeNPs/kg for 8 weeks (N = 9). Data were expressed as Mean ± SEM. Values with a different letter superscript or none indicate significant or insignificant difference between groups. (One-way ANOVA with post-hoc Tukey test, *P* < 0.05; *P* < 0.01; *P* < 0.001).

The bar graphs are shown in Fig. 5. shows the relative expression of proinflammatory and stress genes, including *TNF-α, IL-10, TGF-β1,* and* HSP70*. The data obtained from the gene expression analysis confirmed that no inflammatory response was elicited upon SeNPs supplementation, as reflected by the insignificant difference in expression levels between the control and supplemented fish groups.

### Histopathological examination

**Fig. 6:**
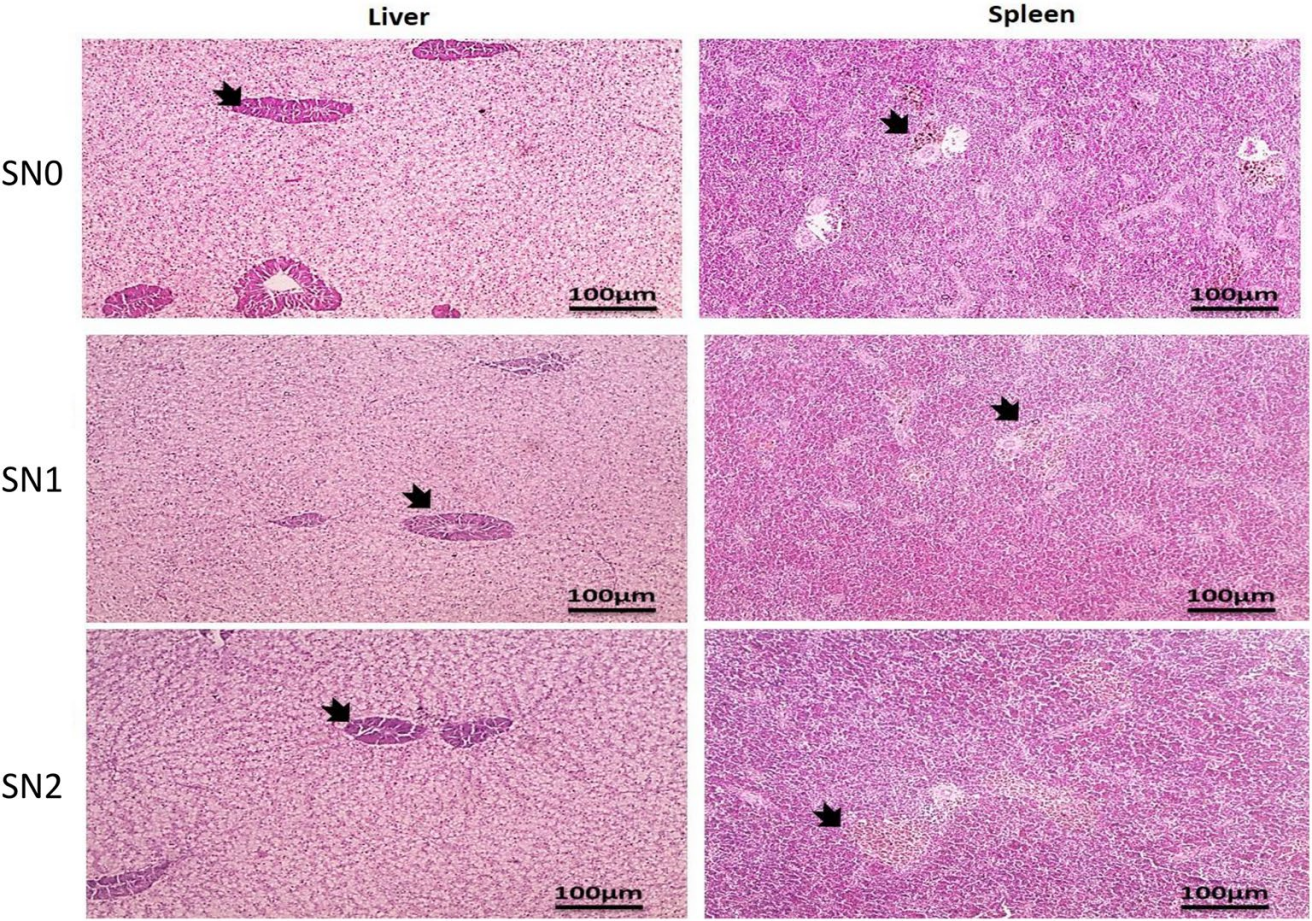
Photomicrograph of H&E-stained sections from liver and spleen of Nile tilapia supplemented with green synthesized selenium nanoparticles (SeNPs) at 0, 0.75, and 1.5 mg SeNPs/kg for 8 weeks. Liver shows normal histological appearance of hepatocytes, sinusoids, hepatopancreas (thick black arrows) in all groups. Spleen shows histological appearance of white pulp and red pulp including melanomacrophage centers (thick black arrows). However, slightly enlarged melanomacrophage centers are observed in group SN2 (thick black arrows) compared to other groups. Low magnification X: 100 bar 100.

The histopathological examination of the photomicrographs of H&E-stained sections from the liver and spleen of the control group and the groups supplemented with SN1 and SN2 are presented in Fig. 6. In the liver sections, a normal histological appearance of hepatocytes, sinusoids, and hepatopancreas was observed in all groups, as indicated by thick black arrows. The histological appearance of white and red pulp, including melanomacrophage centers, is shown in the spleen sections, as indicated by thick black arrows. However, slightly enlarged melanomacrophage centers were observed in the SN2 group compared with the other groups.

## Discussion

Selenium is an essential micronutrient required for normal growth and health of fish. Supplementing fish feed with selenium nanoparticles has recently emerged as a strategy for increasing selenium bioavailability. In this study, *P. boryanum* was utilized as a capping agent to synthesize SeNPs, serving as biological reducing agents for salt ions, leading to their conversion into nanoparticles. Subsequently, these particles were stabilized, as indicated by their zeta potential values^19,32^. Therefore, the eco-friendly approach for producing nano-selenium from microalgal extracts represents an economically feasible method that facilitates the formation of stable selenium nanoparticles^19^.

In the present study, SeNP supplementation did not negatively influence ALT and AST enzyme activity or the protein profile. This result, along with the anti-inflammatory and antioxidant effects of SeNPs were histopathologically evident (as described below). This emphasizes the important role of SeNPs in safeguarding the morphology and structure of liver tissue from toxic damage, with subsequent overall liver function improvement and normal levels of liver enzymes such as AST and ALT preserved. Our data are consistent with those of previous studies^33,34^, emphasizing the safety and physiological compatibility of green-synthesized SeNPs using *Pediastrum boryanum* algae supplementation in Nile tilapia, supporting their potential application in aquaculture without apparent negative effects on liver health or protein metabolism.

Lysozyme (LZM) and IgM showed a dose-dependent increase compared to the control group. Our data coincided with those of other studies on Nile tilapia-fed bio-SeNPs at the same levels and showed a > 1.5 fold-increase in LZM activity^35^. Similarly, a 1.2–1.4-fold increase in LZM activity was observed in Nile tilapia fed Nano-selenium spheres produced by lactic acid bacteria at similar feeding levels^33,34^. On the other hand, Jha et al.^36^ found a 3.6-fold increase in *Cyprinus carpio*-fed polysaccharide-based selenium (AMLP-SeNPs) at a 2 mg/kg diet. IgM levels were improved in a dose-dependent manner (1.5-fold increase), which was similar to the study by Ibrahim et al.^37^, and better than the 1.2-fold increase observed by Ghazi et al.^12^ in Nile tilapia using non-bio-SeNPs. Our findings suggest a key role of SeNPs in controlling different immune cells and altering key immune-related signaling pathways^38^. This involves increasing the production of lymphocyte protein^16^ and boosting the function and proliferation of immune cells, including those that generate lysozyme and IgM antibodies. Thereby improving the overall cellular and humoral responses of the immune system.

The present study found that SeNPs supplementation enhanced antioxidant enzyme activity but did not induce oxidative stress. In the present study, the increased levels of CAT activity were better than those in other studies conducted in Nile tilapia and at comparable SeNPs levels^34–36^. SeNPs led to the enhancement of the glutathione family, including GPx, GSH, and GST, which conformed with the increased level of GSH in the present study. However, the SOD levels showed no statistical changes in our study, which is consistent with previous studies^39–41^. The observed improvement in antioxidant activities could be due to the antioxidant effect of Se, which is involved as a structural component, selenocysteine, in glutathione enzymes^42^, which prohibit cellular membrane peroxidation by catalyzing the removal of reactive oxygen species (ROS) in the fish body^16,43,44^. In addition, SeNPs demonstrated potent antioxidant activity in DPPH free radical-scavenging assays, as evidenced in our recent study^19^. SOD, however, showed a significant increase in some other studies^33,34^, unlike our data, which could be related to the direct action of Se as an antioxidant agent, sparing the SOD level with no change. SeNPs have displayed lower toxicity, as shown in our recent study^19^, which allows for higher dosing and potentially greater antioxidant impact. Furthermore, SeNPs exhibit superior bioavailability in comparison to other selenium forms, as indicated in our earlier study^22^, due to their small size, which enables better cellular uptake and distribution. Using *P. boryanum* microalga as a capping agent to stabilize the SeNPs and prevent agglomeration may offer functional groups for targeted delivery or additional antioxidant effects^45^. Therefore, these enzymes have been noted as indicators of the effects of selenium on antioxidant mechanisms in fish^46^.

*TNF-α* is a pro-inflammatory cytokine that serves as a prognostic marker for changes in immune responses^47^, whereas *HSP70* is a molecular chaperone that protects fish cells from environmental stressors^48^. *IL-10* is an anti-inflammatory cytokine that reduces inflammation and prevents unnecessary T-cell reactions in response to microbial infections, thereby preventing tissue damage and chronic inflammation^49^. *TGF-β1* is a multifunctional cytokine that regulates cellular processes, including growth, differentiation, and immune responses and plays a pivotal role in maintaining tissue homeostasis and in responding to injury or inflammation^49^. As observed, dietary supplementation of SeNPs in Nile tilapia did not induce any inflammatory responses, which is supported by our earlier results of MDA levels and histopathological examination (see below), confirming the absence of excessive ROS, which are the signal inducers for the cascades of pro-inflammatory cytokines through the inhibition of NF-κB activation, a pivotal transcription factor responsible for controlling the production of pro-inflammatory cytokines^50^. Our findings concur with those of previous studies^22,39,41^ that reported insignificant changes in the gene expression of proinflammatory and stress biomarkers. These data suggest that green-synthesized SeNPs from *Pediastrum boryanum* algae maintain body homeostasis and immune regulation without eliciting an inflammatory response and emphasis the role of SeNPs in rebalancing pro- and anti-inflammatory cytokines, leading to a more controlled inflammatory reaction. The data provided in this study were histopathologically evident, and no alterations in liver and spleen tissue architecture were found. Our results corroborate previous studies showing that dietary inclusion of SeNPs at comparable levels maintained liver health^51,52^.

In the present study, an approximately 1.5-fold increase in FW was observed in the supplemented groups compared to that in the control, which is in line with Dawood et al.^33^, who found a positive quadratic influence on fish final weight at similar doses using Nano-selenium spheres produced by lactic acid bacteria (LAB-Se, Lactomicrosel^®^) and of size range 100–500 nm. Jha et al.^36^ observed a significantly higher final weight (2.67-fold) and final length (1.61-fold) in common carp fed *Avicennia marina* leaves polysaccharides-SeNPs (AMLP-SeNPs) of size range 200–700 nm, but at a higher supplemented dose of 2 mg/kg compared with our study. At a comparable dose of 1 mg chemically synthesized SeNPs/kg and of size range 108 ± 11 nm, Nile tilapia showed a 1.33-fold increase, lower than ours, with no alterations in the K factor, SSI, and HSI values^12^. Additionally, Al-Din^53^ reported that adult Nile tilapia-fed SeNPs synthesized via ascorbic acid and of size range 38.45–66.78 nm, at 1 mg/kg, had a 0.93-fold decrease in final weight. Therefore, these discrepancies in the outcomes of growth performance after SeNPs dietary supplementation could be due to interacting factors such as the size of the NPs, the green synthesis methods with algal, plant, or bacterial utilization, and most importantly, the type of trial; in our case, this is a field trial; however, others were mainly laboratory studies. Our results of growth improvement could be attributed to the higher bioavailability of Se using green synthesis methods and its involvement as a precursor of selenoproteins synthesis, which enhances digestive enzymes and intestinal absorption, boosts feed utilization, and enhances growth performance, as evidenced recently in our study focused on the role of SeNPs on intestinal function^22^. Additionally, microalgae have a synergistic role with SeNPs through their antibacterial effect, as reported in our recent study^19^, which inhibits the pathogenic microflora and favors the beneficial microflora with subsequent improvement of nutrient absorption and growth performance.

In conclusion, the findings of this study underscore the potential benefits of incorporating green-synthesized SeNPs into the diet of Nile tilapia (*Oreochromis niloticus*). The study observed that tilapia-fed SeNPs exhibited enhanced growth performance and improved innate immune response and antioxidant enzyme activities. Notably, no signs of oxidative stress or inflammatory changes were observed. Moreover, the dietary inclusion of green-synthesized SeNPs appeared to preserve the health and integrity of the liver and spleen. Collectively, these results provide a compelling argument for applying green-synthesized SeNPs in the formulation of efficient and sustainable feeds to boost the productivity and quality of tilapia aquaculture.

## Supplementary Information

Supplementary Table 1.

Supplementary Information.

## Data Availability

All data supporting the findings of this study are available within the paper.
