# Supplementary material for: Dietary biogenic selenium nanoparticles improve growth and immune-antioxidant indices without inducing inflammatory responses in Nile tilapia
Source: Sci Rep. 2024 Sep 23;14:21990. doi: 10.1038/s41598-024-72022-w (PMC11420227; doi:10.1038/s41598-024-72022-w)
Supplement: Supplementary file 1 — Supplementary Table 1. [file 41598_2024_72022_MOESM1_ESM.docx]

**Supplementary Table 1. Formulation and proximate composition of basal and experimental diets**

| **Ingredients (%)** | **Control** | **SeNPs_0.75_** | **SeNPs_1.5_** |
| --- | --- | --- | --- |
| Yellow corn | 19.5 | 19.5 | 19.5 |
| Soybean meal | 20 | 20 | 20 |
| Fish meal | 20 | 20 | 20 |
| Corn gluten | 3 | 3 | 3 |
| Gelatin | 2 | 2 | 2 |
| Sunflower oil | 3.50 | 3.50 | 3.50 |
| Wheat bran | 30.16 | 30.16 | 30.16 |
| Minerals and vitamins premix | 1 | 1 | 1 |
| Salt | 0.30 | 0.30 | 0.30 |
| Vitamin C | 0.12 | 0.12 | 0.12 |
| Dicalcium phosphate | 0.10 | 0.10 | 0.10 |
| Methionine | 0.32 | 0.32 | 0.32 |
| SeNPs (mg/kg) | 0 | 0.75 | 1.5 |
| **Proximate analysis (% dry matter basis)** | | | |
| Crude Protein* | 32.04 | 32.04 | 32.04 |
| Lipid* | 7.06 | 7.06 | 7.06 |
| Ca* | 1.17 | 1.17 | 1.17 |
| P* | 0.53 | 0.53 | 0.53 |
| DE (Digestable Energy)** (kcal/kg) | 3016 | 3016 | 3016 |
| Se content (mg/Kg) | 0.2 | 0.75 | 1.5 |

^a^The levels of micronutrients and vitamins for tilapia are covered by supplementation with trace minerals and vitamin premixes as recommended by NRC (2011). Vitamins premix (IU or mg/kg diet); vit. A 5000, Vit.D3 1000, vit. E 20, vit. k3 2, vit. B1 2, vit. B2 5, vit. B6 1.5, vit. B12 0.02, Pantothenic acid 10, Folic acid 1, Biotin 0.15, Niacin 30. Mineral mixture (mg/kg diet); Fe 40, Mn 80, Cu 4, Zn 50, I 0.5, Co 0.2 & Se 0.2. *Analysed. **DE calculated according to [Jobling ^54^](#_ENREF_54). The Gross energy calculated according to NRC (2011), as follows: CP×5.64+EE×9.44+NFE×4.11; whereas [Nitrogen free extract (NFE) = [100-(CP+ EE+ CF+ Ash)]. The DE was calculated according to Jobling (1983) as follows: digestible energy= gross energy × 0.75.
