# Supplementary material for: Dietary biogenic selenium nanoparticles improve growth and immune-antioxidant indices without inducing inflammatory responses in Nile tilapia
Source: Sci Rep. 2024 Sep 23;14:21990. doi: 10.1038/s41598-024-72022-w (PMC11420227; doi:10.1038/s41598-024-72022-w)
Supplement: Supplementary file 2 — Supplementary Information. [file 41598_2024_72022_MOESM2_ESM.pdf]

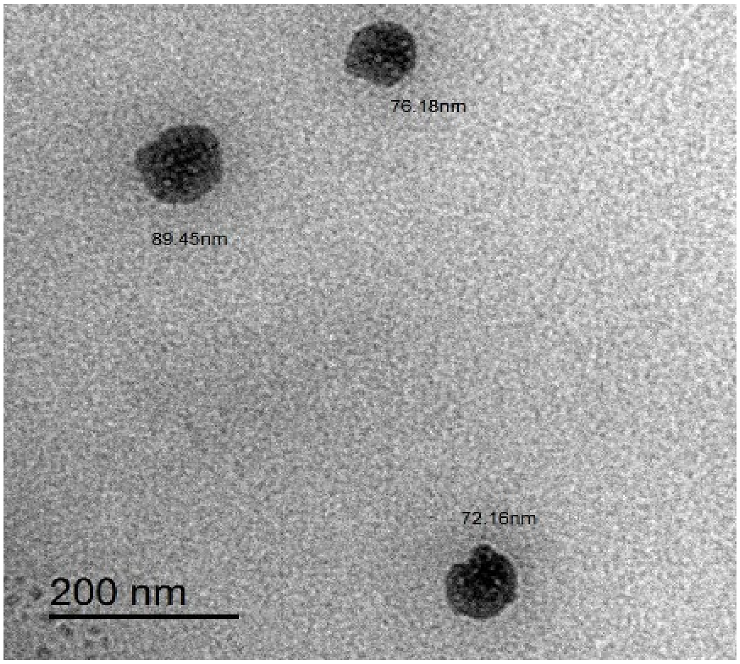

TEM micrographs showing the Bio-SeNPs green synthesized using *P. boryanum* algal extract.

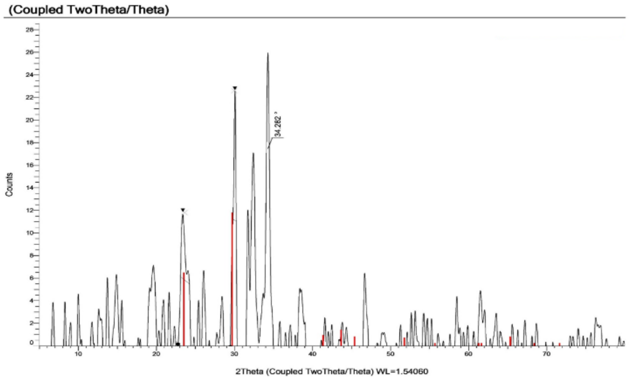

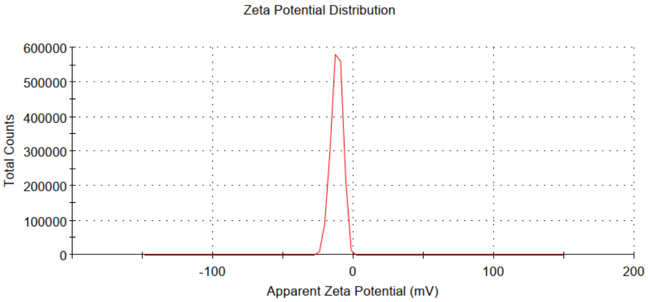

XRD of Selenium nanoparticles	Zeta potential for the Bio-SeNPs green synthesized from *P. boryanum* algal extract.
